# Dilated Left Ventricular End-Diastolic Diameter Is a New Risk Factor of Acute Kidney Injury Following Coronary Angiography

**DOI:** 10.3389/fcvm.2022.827524

**Published:** 2022-03-28

**Authors:** Qiang Li, Shiqun Chen, Haozhang Huang, Weihua Chen, Liwei Liu, Bo Wang, Wenguang Lai, Shixin Yi, Ming Ying, Ronghui Tang, Zhidong Huang, Jiayi Deng, Jiyan Chen, Jin Liu, Yong Liu

**Affiliations:** ^1^Department of Cardiology, Guangdong Cardiovascular Institute, Guangdong Provincial People's Hospital, Guangdong Academy of Medical Sciences, Guangzhou, China; ^2^Department of Guangdong Provincial Key Laboratory of Coronary Heart Disease Prevention, Guangdong Cardiovascular Institute, Guangdong Provincial People's Hospital, Guangdong Academy of Medical Sciences, Guangzhou, China; ^3^Longyan First Affiliated Hospital of Fujian Medical University, Longyan, China; ^4^School of Biology and Biological Engineering, Guangdong Provincial People's Hospital, South China University of Technology, Guangzhou, China; ^5^Department of Ultrasound Imaging, Yunnan Fuwai Cardiovascular Hospital, Kunming, China; ^6^School of Medicine, Guangdong Provincial People's Hospital, South China University of Technology, Guangzhou, China

**Keywords:** acute kidney injury, dilated left ventricular end-diastolic diameter, predictor, coronary angiography, coronary artery disease

## Abstract

**Purpose:**

Left ventricular end-diastolic diameter (LVEDD) is a common indicator in echocardiogram, and dilated LVEDD was correlated with left ventricular insufficiency. However, it is uncertain whether dilated LVEDD is associated with increasing the risk of contrast-associated acute kidney injury (CA-AKI) in patients with coronary artery disease (CAD).

**Patients and Methods:**

We enrolled 8,189 patients with CAD undergoing coronary angiography (CAG) between January 2007 and December 2018. Patients were divided into two groups according to the LVEDD length (normal LVEDD: men: LVEDD ≤56 mm, women: LVEDD ≤51 mm; dilated LVEDD: men: LVEDD >56 mm, women: LVEDD >51 mm). The endpoints were CA-AKI^0350^ and CA-AKI^0525^ (CA-AKI^0350^: an increase in the serum creatinine (Scr) level by >0.3 mg/dl or >50% within the first 48 h after CAG; CA-AKI^0525^: an absolute Scr increase ≥ 0.5 mg/dl or a relative increase ≥ 25% within 72 h after contrast medium exposure). In-hospital dialysis, 30-day mortality, and 1-year mortality were contained as well. Univariate and multivariable logistic regressions were used to assess the association between LVEDD and CA-AKI.

**Results:**

Among 8,189 participants (men: 76.6%, mean age: 64.4 ± 10.7 years), 1,603 (19.6%) presented with dilated LVEDD. In addition, the dilated LVEDD group indicated an elevation of CA-AKI^0350^ (12.4 vs. 6.2%, *p* < 0.001) and CA-AKI^0525^ (15.0 vs. 8.8%; *p* < 0.001) when compared with the normal group. According to multivariable logistic analysis, dilated LVEDD was an independent predictor of CA-AKI^0350^ [adjusted odds ratio (aOR): 1.31; 95% confidence interval (CI): 1.06–1.61, *p* = 0.010) and CA-AKI^0525^ (aOR: 1.32; 95% CI: 1.04–1.67; *p* = 0.020).

**Conclusion:**

In conclusion, these results demonstrated that the dilated LVEDD was a significant and independent predictor of CA-AKI following CAG in patients with CAD. Further verifications are needed to verify the association between LVEDD and CA-AKI.

## Introduction

Contrast-associated acute kidney injury (CA-AKI) is a common complication following coronary angiography (CAG) (1), which is associated with poor outcomes, including dialysis, longer duration of hospitalization, mortality, and increased health-care costs (2–5).

Left ventricular end-diastolic diameter (LVEDD) is a common and important indicator in echocardiogram, which reflects the size of cardiac as well as left ventricular function. It is associated with progressive left ventricular insufficiency (6–9). At the same time, dilated LVEDD is linked to a high risk for heart failure and cardiovascular outcomes (10, 11).

Previous studies have shown that cardiac dysfunction is closely related to the occurrence and development of CA-AKI (12). As a clinically significant index used to assess cardiac function, left ventricular ejection fraction (LVEF) has been proved an independent risk factor for postoperative CA-AKI (13, 14). Similarly, Liu et al. have demonstrated that left ventricular end-diastolic pressure (LVEDP), an indicator of cardiac function, is independently associated with increased risk of CA-AKI among patients undergoing CAG and percutaneous coronary intervention (15). Monitoring hemodynamic parameters, such as LVEDP, is an invasive and expensive test. LVEDD is a novel and convenient imaging predicator reflecting cardiac function, but it has not established a direct relationship between LVEDD and the occurrence of CA-AKI.

Therefore, the aim of our study was to investigate the association of LVEDD levels with CA-AKI in a large registry of patients with coronary artery disease (CAD), who underwent CAG.

## Materials and Methods

### Study Design and Population

**Figure 1:**
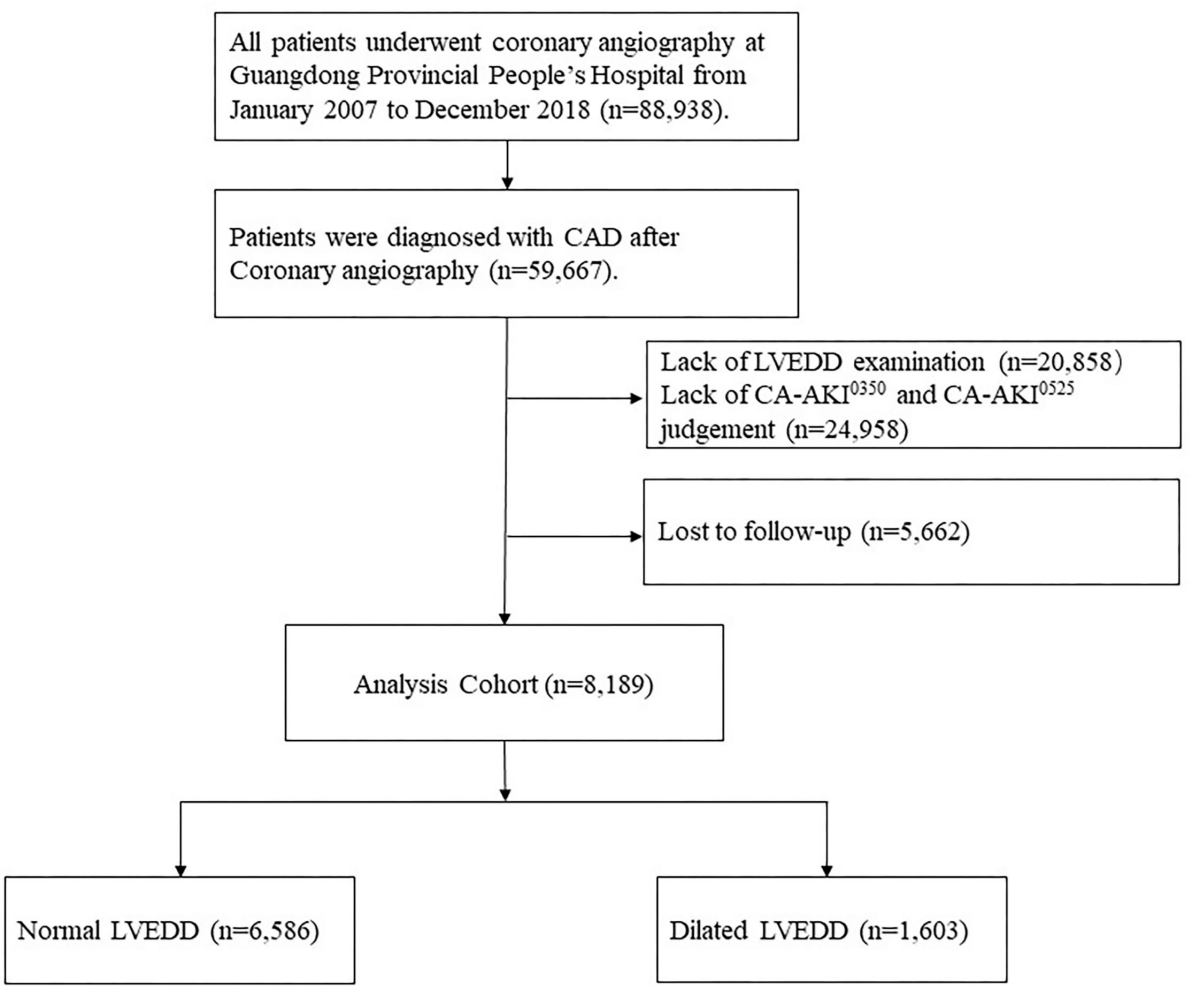
Study flowchart.

The data of this cohort was from the registry of Cardiorenal Improvement (CIN) study (ClinicalTrials.gov NCT04407936) at Guangdong Provincial People's Hospital in China. A total of 8,189 patients with CAD, who underwent CAG between January 2007 and December 2018, were included in the final analysis after excluding 51,478 patients for the following reasons: (1) lack of LVEDD examination (*n* = 20,858); (2) lack of judgment of CA-AKI^0350^ and CA-AKI^0525^(*n* = 24,958); (3) lack of follow-up information (*n* = 5,662) (Figure 1). This study was conducted in accordance with the Declaration of Helsinki and was approved by the Research Ethics Committee of Guangdong Provincial People's Hospital (No. GDREC2019555H).

### Baseline Data Collection

The study data were derived from the Clinical Management System of the Guangdong Provincial People's Hospital's electronic health record, which contained details regarding demographics, medical history, laboratory tests, medications, and other clinical data. Senior cardiologists were responsible for the data quality control and periodical data verification. During the study period, the information on death event and date for each patient was retrieved from the Guangdong Public Security System, which was linked to CIN dataset by a unique identification number. CAG or percutaneous coronary intervention was performed according to the standard clinical practice guidelines (16–18).

### LVEDD Measurement

Echocardiography was performed by trained cardiologists for all patients at the time of admission. LVEDD was measured using an M-mode scan of the parasternal long-axis view with a 2D image. When the M-mode cursor could not be aligned perpendicularly to the left ventricular long axis, LVEDD was measured directly on 2D images (19).

### Study Endpoint and Definitions

The primary outcome was CA-AKI^0350^, defined as an absolute increase in serum creatinine (Scr) ≥ 0.3 mg/dl or a relative increase in Scr levels ≥ 50% within 48 h after contrast medium exposure (20). The secondary clinical outcomes included CA-AKI^0525^ (defined as an absolute increase in Scr levels ≥ 0.5 mg/dl or a relative increase in Scr levels ≥ 25% within 72 h after contrast medium exposure) (21), in-hospital dialysis, 30-day mortality, and 1-year mortality. CAD was confirmed by CAG and discriminated according to the 10th Revision Codes of the International Classification of Diseases (ICD-10; I20.xx–I25.xx, I50.00001, and I91.40001 et al.). Estimated glomerular filtration rate (eGFR) was estimated by the Chronic Kidney Disease Epidemiology Collaboration (CKD-EPI) equation (22). CKD was defined as eGFR < 60 ml/min/1.73 m^2^. Anemia was defined as a baseline hematocrit value < 39% for men and <36% for women, according to the World Health Organization (WHO) criteria (23). The echocardiography examination was used to evaluate the LVEF and heart failure with reduced ejection fraction (HFrEF), which was defined as LVEF<40% (24).

### Statistical Analysis

To investigate the relationship of elevated LVEDD levels for CA-AKI in patients with CAD, men and women were divided into normal LVEDD group and dilated LVEDD group (men: LVEDD ≤56, and>56 mm; women: LVEDD ≤51, and>51 mm, respectively) (25). We reported descriptive statistics by means (SD), median [interquartile range (IQR)], or number and percentage as and when it was needed. Categorical variables were compared by Pearson chi-square test, and continuous variables by *t*-test. The association between different LVEDD levels and CA-AKI was tested by univariable and multivariable logistic regression. To investigate the relationship between LVEDD and LVEF, scatter plot and curve fitting were performed as shown in Supplementary Figure 1. All analyses were performed using R software (version 4.0.3; R Foundation for Statistical Computing, Vienna, Austria). A two-sided *p* < 0.05 indicated significance for all analyses.

## Results

### Clinical Characteristics

After screening, a total of 8,189 CAD patients, who underwent CAG from January 2007 to December 2018, were included in this study. The mean age was (64.4 ± 10.7) years, and 6,273 patients (76.6%) were men. Patients were divided into two groups: 1,603 (19.6%) patients with dilated LVEDD (men: LVEDD>56 mm; women: LVEDD>51 mm), and 6,586 (80.4%) patients with normal LVEDD (men: LVEDD ≤56 mm; women: LVEDD ≤51 mm). Compared with the normal LVEDD group, the dilated LVEDD group was more likely to present with anemia, diabetes mellitus (DM), and reduced LVEF (< 40%), and had worse renal function. All of the baseline clinical characteristics of the patients are shown in Table 1.

**Table 1:** Baseline characteristics of the patients.

**Characteristic**	**Overall**	**Normal LVEDD**	**Dilated LVEDD**	***P*-value**
	**(*n* = 8,189)**	**(*n* = 6,586)**	**(*n* = 1,603)**	
**Demographic**				
Age, years	64.44 ± 10.73^*^	64.43 ± 10.78^*^	64.49 ± 10.52^*^	0.822
Age>75, *n* (%)	1,534 (18.73)	1,232 (18.71)	302 (18.84)	0.931
Male, *n* (%)	6,273 (76.60)	5,066 (76.92)	1,207(75.30)	0.179
**Medical history**				
AMI, *n* (%)	2,067 (25.27)	1,697 (25.80)	370 (23.08)	0.027
HT, *n* (%)	4,969 (60.74)	4,028 (61.23)	941 (58.70)	0.067
DM, *n* (%)	2,535 (30.99)	1,952 (29.67)	583 (36.37)	<0.001
CHF, *n* (%)	1,374 (16.79)	807 (12.26)	567 (35.37)	<0.001
HFrEF, *n* (%)	1,211 (14.86)	383 (5.85)	828 (51.72)	<0.001
CKD, *n* (%)	432 (5.28)	293 (4.45)	139 (8.67)	<0.001
Anemia, *n* (%)	3,468 (42.74)	2,695(41.30)	773(48.65)	<0.001
PCI, *n* (%)	6,318 (77.15)	5,176 (78.59)	1,142 (71.24)	<0.001
**Laboratory test**				
Glu, mmol/L	7.59 ± 3.58^*^	7.50 ± 3.52^*^	7.96 ± 3.81^*^	<0.001
HbA1c, %	6.63 ± 1.47^*^	6.59 ± 1.46^*^	6.77 ± 1.48^*^	<0.001
HDLC, mmol/L	0.97 ± 0.25^*^	0.97 ± 0.25^*^	0.94 ± 0.25^*^	<0.001
LDLC, mmol/L	2.80 ± 0.99^*^	2.80 ± 0.97^*^	2.79 ± 1.04^*^	0.513
HGB, g/L	129.06 ± 18.80^*^	129.83 ± 18.06^*^	125.89 ± 21.28^*^	<0.001
eGFR, ml/min/1.73 m^2^	67.73 ± 24.73	69.88 ± 24.10	58.91 ± 25.32	<0.001
LVEF, %	56.02 ± 13.42^*^	59.75 ± 10.66^*^	40.76 ± 12.74^*^	<0.001
CMV, ml	154.88 ± 85.74^*^	156.24 ± 85.22^*^	149.33 ± 87.65^*^	0.004
CMV>200, *n* (%)	1,243 (15.59)	1,008 (15.74)	235 (15.00)	0.493
**Medication**				
Antiplatelet, *n* (%)	7,633 (95.44)	6,196 (96.03)	1,437 (92.95)	<0.001
ACEI/ARB, *n* (%)	4,000 (50.01)	3,246 (50.31)	754 (48.77)	0.290
Beta-blockers, *n* (%)	6,554 (81.95)	5,282 (81.87)	1,272 (82.28)	0.734
Statins, *n* (%)	7,550 (94.40)	6,143 (95.21)	1,407 (91.01)	<0.001
Diuretics, *n* (%)	2,152 (26.91)	1,259 (19.51)	893 (57.76)	<0.001

* *Data expressed as mean ± standard deviation*.

*PCI, percutaneous coronary intervention; AMI, acute myocardial infarction; HT, hypertension; DM, diabetes mellitus; CHF, congestive heart failure; HFrEF, Heart failure with reduced ejection fraction; CKD, chronic kidney disease; Glu, glucose; HbA1c, hemoglobin A1c; HDLC, high-density lipoprotein cholesterol; LDLC, low-density lipoprotein cholesterol; HGB, hemoglobin; eGFR, estimated glomerular filtration rate; LVEF, left ventricular ejection fraction; CMV, contrast medium volume; ACEI/ARB, angiotensin-converting enzyme inhibitor/angiotensin receptor blocker*.

### Incidence of CA-AKI and Clinical Outcomes

Totally, 607 (7.4%) patients fulfilled the diagnostic criteria for CA-AKI^0350^, and the incidence of CA-AKI^0350^ among the dilated LVEDD group was apparently higher than the normal group (12.4 vs. 6.2%; *p* < 0.001, Table 2). A similar result was observed in the incidence of CA-AKI^0525^ (15.0 vs. 8.8%; *p* < 0.001). The in-hospital dialysis rate, and 30-day and 1-year mortalities were all significantly higher in patients with dilated LVEDD when compared with those with normal LVEDD(4.9 vs. 1.9%, 2.4 vs. 1.2%, 9.7 vs. 4.1%, respectively; all *p* < 0.001).

**Table 2:** Contrast-associated acute kidney injury (CA-AKI) incidence and clinical outcomes.

**Events**	**Overall**	**Normal LVEDD**	**Dilated LVEDD**	***P*-value**
	**(*n* = 8,189)**	**(*n* = 6,586)**	**(*n* = 1,603)**	
CA-AKI^0350^, *n* (%)	607 (7.41)	409 (6.21)	198 (12.35)	<0.001
CA-AKI^0525^, *n* (%)	818 (9.99)	577 (8.76)	241 (15.03)	<0.001
In-hospital dialysis, *n* (%)	203 (2.48)	124 (1.88)	79 (4.93)	<0.001
30-day death, *n* (%)	117 (1.43)	78 (1.18)	39 (2.43)	<0.001
1-year death, *n* (%)	424 (5.18)	269 (4.08)	155 (9.67)	<0.001

*CA-AKI^0350^: an absolute Scr increase ≥ 0.3 mg/dL or a relative increase in serum creatinine ≥ 50% within 48 h after contrast medium exposure*.

*CA-AKI^0525^: an absolute Scr increase ≥ 0.5 mg/dL or a relative increase in serum creatinine ≥ 25% within 72 h after contrast medium exposure*.

### Association of LVEDD With AKI

Univariate logistic regression analysis indicated that the dilated LVEDD [odds ratio (OR): 2.1; 95% confidence interval (CI): 1.78–2.54; *p* < 0.001, Supplementary Table 1] was significantly associated with CA-AKI^0350^ after CAG. When using the criteria of CA-AKI^0525^, similar result was found (OR: 1.8; 95% CI: 1.57–2.16; *p* < 0.001, Supplementary Table 1). In this study, age, anemia, CKD, DM, and HFrEF were also closely related to CA-AKI^0350^ and CA-AKI^0525^ (all *p* < 0.001, Supplementary Table 1). After adjusting for related risk factors (age, gender, hypertension, acute myocardial infarction (AMI), CKD, HFrEF, DM, contrast medium volume (CMV) >200, and diuretics), dilated LVEDD [adjusted odds ratio (aOR): 1.3; 95% CI: 1.06–1.61; *p* = 0.010, Table 3) remained significantly associated with CA-AKI^0350^. Similar results were obtained in CA-AKI^0525^ (aOR: 1.32; 95% CI: 1.04–1.67; *p* = 0.020, Table 3).

**Table 3:** Multivariable regression analysis of CA-AKI in different definition.

**Variables**	**CA-AKI** ^**0350**^		**CA-AKI** ^**0525**^	
	**OR (95% CI)**	***P*-Value**	**OR (95% CI)**	***P*-Value**
Dilated vs. normal^a^	1.31 (1.06–1.61)	0.010	1.32 (1.04–1.67)	0.020
Age	1.01 (1.01–1.01)	<0.001	1.02 (1.01–1.03)	<0.001
Gender	1.58 (1.33–1.88)	<0.001	1.23 (1.00–1.51)	0.052
AMI	1.11 (0.94–1.32)	0.205	0.81 (0.65–1.01)	0.069
HT	1.01 (0.85–1.19)	0.938	1.02 (0.83–1.25)	0.869
DM	1.11 (0.94–1.32)	0.205	1.21 (0.99–1.46)	0.056
Anemia	1.36 (1.15–1.60)	<0.001	1.46 (1.21–1.77)	<0.001
HFrEF	1.53 (1.21–1.94)	<0.001	1.48 (1.19–1.83)	<0.001
CKD	2.40 (1.99–2.90)	<0.001	0.99 (0.84–1.17)	0.926
CMV>200	1.22 (0.99–1.50)	0.063	1.43 (1.13–1.81)	0.003
Diuretics	1.96 (1.65–2.34)	<0.001	1.84 (1.50–2.25)	<0.001

*a: Dilated LVEDD compared with Normal LVEDD1*.

*CA-AKI^0350^: an absolute Scr increase ≥ 0.3 mg/dL or a relative increase in serum creatinine ≥ 50% within 48 h after contrast medium exposure*.

*CA-AKI^0525^: an absolute Scr increase ≥ 0.5 mg/dL or a relative increase in serum creatinine ≥ 25% within 72 h after contrast medium exposure*.

*LVEDD, left ventricular end-diastolic diameter; AMI, acute myocardial infarction; HT, hypertension; DM, diabetes mellitus; HFrEF, Heart failure with reduced ejection fraction; CKD, chronic kidney disease; CMV, contrast medium volume*.

## Discussion

To our knowledge, this is the first cohort study that investigates the relationship between LVEDD and CA-AKI in patients with CAD. The results of our study showed that dilated LVEDD was markedly related to the incidence of CA-AKI in patients with CAD, regardless of reduced LVEF.

A number of risk factors, such as laboratory examinations (Scr, age, and gender), comorbidities, and medications, have been confirmed to be associated with CA-AKI (26, 27). Some risk models, such as Merhan score and age, creatinine, and ejection fraction (ACEF) score, were commonly used to assess the risk of CA-AKI in clinical practice (28, 29). However, these methods were reviewed and contain multiple variables, which limits their clinical use. Therefore, it is necessary to explore a variable that is common and easy to access to determine the risk of AKI. The echocardiogram is readily available and widely used in the diagnosis of cardiovascular disease.

Left ventricle structural dilation used to be considered as the morphologic substrate of congestive heart failure (CHF) for the first time by a German pathologist Linzbach AJ (30). Using left ventricular dimensions in the risk stratification of patients will aid in the better identification of patients at risk for heart failure and mortality (11). Many studies have indicated that LVEDD dilation is implicated in the development of progressive left ventricular insufficiency and CHF (7, 9). Moreover, dilated LVEDD is associated with major adverse cardiovascular events in patients with heart failure and left ventricular systolic insufficiency (10). Moreover, it was also reported that left ventricular dilation could be used for identifying cardiac death and serious ventricular arrhythmias independently in left ventricular systolic insufficiency (6, 8). Our study suggests that an adjusted large LVEDD is still an independent factor associated with higher incidence of CA-AKI, even after adjusting for ejection fraction <40, which manifests left ventricular systolic insufficiency. It confirms that dilated LVEDD is an independent predictor for CA-AKI without relying on LVEF. Likewise, our study showed that there was no clear relationship between LVEDD and LVEF. The possible reason why related adverse events were caused by left ventricular dilation is that increased ventricular wall stress in the dilated ventricle causes a mismatch in cardiac afterload, which leads to hemodynamic changes and further cardiac and renal adverse events, such as CA-AKI.

Previous studies also have found that LVEDD level is a risk factor for mortality in patients with or without heart failure. Seko et al. retrospectively analyzed data from 4,444 patients, who underwent echocardiography, and found that LVEDD had a deleterious impact on long-term mortality after adjusting for age and body size (31). The conclusion was similar to the study conducted by Kajimoto K et al., which indicated that after adjustment for multiple comorbidities, men with an LVEDD of 61 mm had a significantly higher risk of the outcomes than men with an LVEDD <54 mm, although this conclusion was weakened among women (32). Our study also found that the group with higher LVEDD had worse outcome events and had a significantly higher incidence of CA-AKI than the normal group.

This study has several important clinical significances and research implications. As it was described above, LVEDD was reported to be an important factor of cardiac function. Our results demonstrated that dilated LVEDD was an independent predictor of CA-AKI among patients with CAD, even after adjusting for potential confounding factors. The outcome demonstrates that we should not only focus on LVEF in echocardiogram but also LVEDD. A routine pre-procedural LVEDD measurement may provide useful information for the cardiologist to identify the high risk for the incidence of AKI. The degree of prediction of AKI can also be improved by combining known risk factors. More attention should be given to patients with dilated LVEDD.

### Limitations

This study has several limitations. First, this observational cohort was conducted in a single center that is located in southern China. However, Guangdong Provincial People's Hospital is the largest cardiovascular center in southern China. Moreover, the sample size included in the study is large enough and patients come from different regions, which provide the results with a certain degree of universality. Second, the velocity and volume of preoperative hydration may influence the incidence of CA-AKI, but they were unrecorded. Third, there are some confounding factors that have not been taken into consideration due to the lack of variables, such as body mass index (BMI), smoking, and other influencing factors. However, special attention was given to avoid biases by adjusting the results in accordance with co-morbidities and clinically relevant in-hospital events. Finally, our database lacks information on hemodynamic disturbances, such as cardiogenic shock or the use of mechanical circulatory support, and coronary-artery-bypass-grafting (CABG) incidence following CAG. Therefore, we may fail to consider the impact of hemodynamic disturbances and post-contrast CABG on the results.

## Conclusion

Our study found that dilated LVEDD may be remarkably related to an increased risk of CA-AKI following CAG in patients with CAD. LVEDD is an easily obtained indicator in echocardiogram, which can be performed before CAG to identify the risk of CA-AKI in patients with CAD. Precautionary measures should be taken in the perioperative period for patients with dilated LVEDD, who have a high risk for CA-AKI.

## Data Availability Statement

The datasets presented in this study can be found in online repositories. The names of the repository/repositories and accession number(s) can be found in the article/Supplementary Material.

## Ethics Statement

The studies involving human participants were reviewed and approved by Research Ethics Committee of Guangdong Provincial People's Hospital. The patients/participants provided their written informed consent to participate in this study.

## Author Contributions

YL, JL, and JC contributed to supervision, mentorship, research idea, and study design. QL, SC, HH, WC, BW, WL, SY, LL, MY, RT, ZH, and JD contributed to data acquisition. JL and YL contributed to data analysis/interpretation. SC and QL contributed to statistical analysis. All authors contributed important intellectual content during manuscript drafting or revision and accepts accountability for the overall work by ensuring that questions pertaining to the accuracy or integrity of any portion of the work are appropriately investigated and resolved.

## Funding

This study was supported by Guangdong Provincial Science and Technology Project (2020B1111170011); by Guangdong Provincial Science and Technology Project (KJ022021049); by Beijing Lisheng Cardiovascular Health Foundation (No. LHJJ20141751); by National Key Research and Development Program Grants of China (2016YFC1301202); and by Guangdong Provincial Key Laboratory of Coronary Heart Disease Prevention (No. 2017B030314041). The funders had no role in the study design, data collection and analysis, decision to publish, or preparation of the manuscript. The work was not funded by any industry sponsors.

## Conflict of Interest

The authors declare that the research was conducted in the absence of any commercial or financial relationships that could be construed as a potential conflict of interest.

## Publisher's Note

All claims expressed in this article are solely those of the authors and do not necessarily represent those of their affiliated organizations, or those of the publisher, the editors and the reviewers. Any product that may be evaluated in this article, or claim that may be made by its manufacturer, is not guaranteed or endorsed by the publisher.
