# Supplementary material for: Dilated Left Ventricular End-Diastolic Diameter Is a New Risk Factor of Acute Kidney Injury Following Coronary Angiography
Source: Front Cardiovasc Med. 2022 Mar 28;9:827524. doi: 10.3389/fcvm.2022.827524 (PMC8996253; doi:10.3389/fcvm.2022.827524)
Supplement: Supplementary file 1 [file Data_Sheet_1.docx]

**Supplementary Table1. Univariate logistic regression analysis of the association between LVEDD and CA-AKI in different definition.**

| **Variables** | **CA-AKI^0350^** | | | **CA-AKI^0525^** | |
| --- | --- | --- | --- | --- | --- |
|  | OR (95% CI) | P-Value |  | OR (95% CI) | P-Value |
| Dilated vs Normal^a^ | 2.13 (1.78-2.54) | <0.001 |  | 1.84 (1.57-2.16) | <0.001 |
| Age | 1.04 (1.03-1.05) | <0.001 |  | 1.03 (1.02-1.03) | <0.001 |
| Gender | 1.38 (1.15-1.66) | 0.001 |  | 1.66 (1.42-1.94) | <0.001 |
| AMI | 0.90 (0.74-1.09) | 0.270 |  | 1.05 (0.89-1.24) | 0.534 |
| HT | 1.40 (1.17-1.67) | <0.001 |  | 1.14 (0.98-1.32) | 0.095 |
| DM | 1.60 (1.35-1.89) | <0.001 |  | 1.31 (1.12-1.52) | 0.001 |
| Anemia | 2.23 (1.89-2.65) | <0.001 |  | 1.65 (1.43-1.91) | <0.001 |
| HFrEF | 2.26 (1.87-2.74) | <0.001 |  | 1.88 (1.58-2.24) | <0.001 |
| CKD | 3.47 (2.93-4.12) | <0.001 |  | 1.41 (1.22-1.64) | <0.001 |
| CMV＞200 | 1.25 (1.00-1.55) | 0.045 |  | 1.13 (0.92-1.37) | 0.229 |

**a:** Dilated LVEDD compared with Normal LVEDD.

**CA-AKI^0350^:** an absolute Scr increase ≥ 0.3 mg/dL or a relative increase in serum creatinine ≥ 50% within 48 hours after contrast medium exposure.

**CA-AKI^0525^:** an absolute Scr increase ≥ 0.5 mg/dL or a relative increase in serum creatinine ≥ 25% within 72 hoaurs after contrast medium exposure.

**Abbreviation:** LVEDD, left ventricular end-diastolic diameter; AMI, acute myocardial infarction; HT, hypertension; DM, diabetes mellitus; HFrEF, heart failure with reduced ejection fraction; CKD, chronic kidney disease; CMV, contrast medium volume.

**Supplementary Figure.1 Correlation between LVEDD and LVEF.**


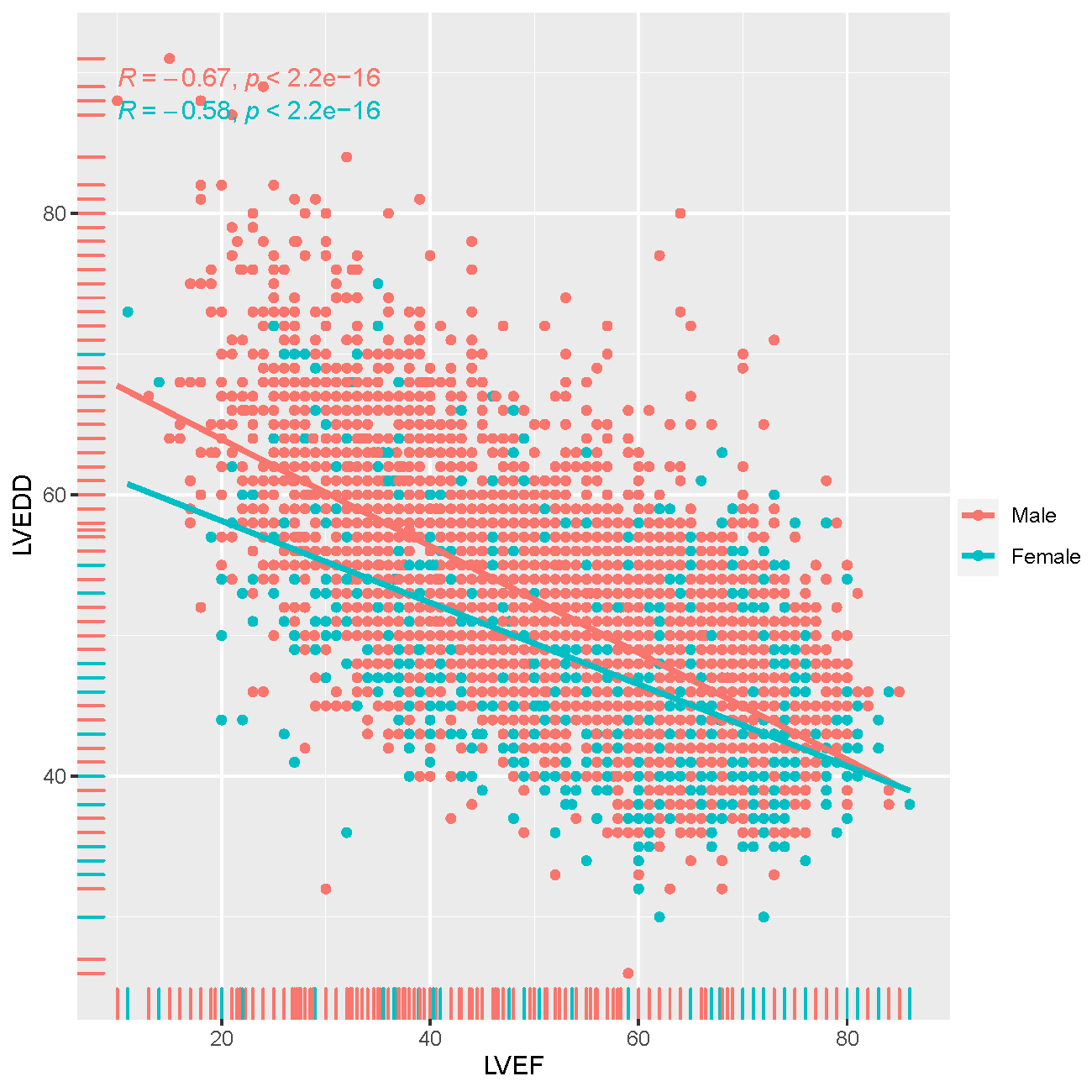


**Abbreviation:** LVEDD, left ventricular end-diastolic diameter; LVEF, left ventricular ejection fraction.
